# Taking cohesin and condensin in context

**DOI:** 10.1371/journal.pgen.1007118

**Published:** 2018-01-25

**Authors:** Kobe C. Yuen, Jennifer L. Gerton

**Affiliations:** 1 Stowers Institute for Medical Research, Kansas City, Missouri, United States of America; 2 Department of Oncology Biomarker Development, Genentech, Inc., South San Francisco, California, United States of America; 3 Department of Biochemistry and Molecular Biology, University of Kansas School of Medicine, Kansas City, Kansas, United States of America; 4 University of Kansas Cancer Center, Kansas City, Kansas, United States of America; Harvard Medical School, UNITED STATES

## Abstract

Structural maintenance of chromosome (SMC) protein complexes, including cohesin and condensin, are increasingly being recognized for their important role in cancer and development, making it critical that we understand how these evolutionarily conserved multi-subunit protein complexes associate with and organize the genome. We review adaptor proteins for SMC complexes and how these adaptors may capture SMC complexes following loop extrusion to provide a framework for chromosome organization.

## SMC complexes have ancient origins and share structural similarities

Condensin is an evolutionarily conserved multi-subunit protein complex critical for chromosome maintenance. Although condensin was first discovered in bacteria in which it is important for the transmission of the bacterial chromosome [1], it was Hirano who first suggested the function of the eukaryotic condensin complex from biochemical characterization [2]. Purified condensin from *Xenopus* egg extracts caused a fuzzy mass of sperm chromatin to visibly condense into thread-like structures. Condensin is now recognized as a member of the SMC family, which includes cohesin, Smc5/6, and the dosage compensation complex (DCC) in *Caenorhabditis elegans*. These protein complexes have been studied for their roles in organizing and segregating chromosomes.

**Fig 1:**
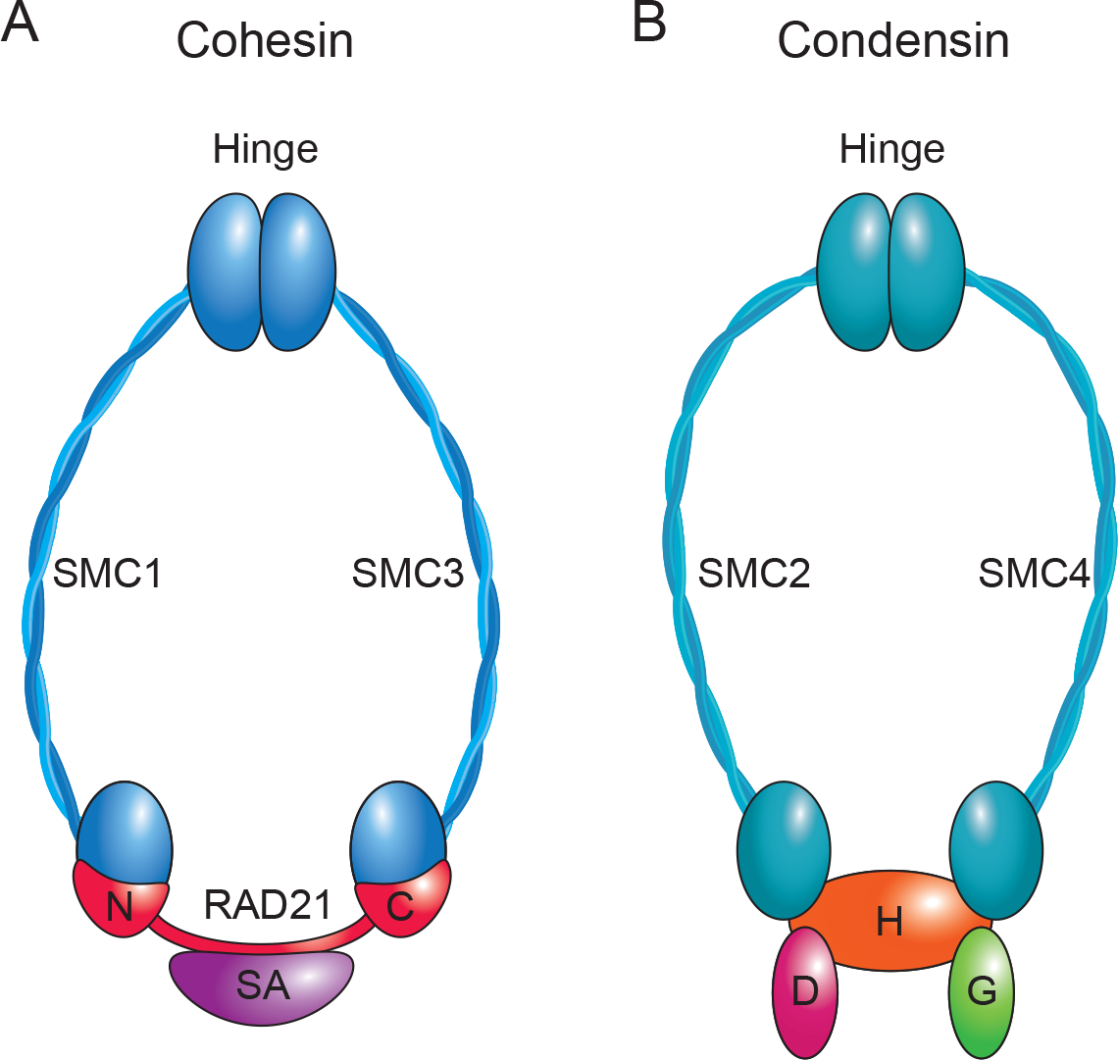
SMC complexes form ring-like structures. A cartoon of a cohesin (A) and condensin (B) protein complex shows the long coiled-coil SMC arms of the ring (blue; SMC1 and SMC3 for cohesin, SMC2 and SMC4 for condensin), as well as the subunits that interact at the head domains (N and C termini of RAD21 and SA for cohesin; Condensin-Associated Proteins CAPH2, CAPD3, and CAPG2 for condensin). In particular, the RAD21 and CAPH2 subunits (red, kleisin) are important for closing the ring at the SMC head domains. The interaction between the hinge domains of the SMC subunits of cohesin may serve as an entry gate for DNA into the ring, and the SMC3–RAD21 interface may serve as an exit gate. Cartoon adapted from [6]. SA, stromal antigen; SMC, structural maintenance of chromosome.

SMC complexes, so named because they all contain SMC subunits, share certain structural features. For example, multiple subunits come together to form a ring-like structure (Fig 1). The SMC subunits have coiled coils that hinge or fold in the middle to make the long “arms” of the complex (approximately 50 nm, blue). For cohesin in yeast, the hinge domains interact to potentially form an entry gate for DNA into the ring. The N and C termini of each SMC monomer peptide come together to form head regions that have ATPase activity [3]. These head domains of the SMC subunits interact with additional subunits, in particular the kleisin subunit (red, RAD21 in cohesin and condensin-associated protein H2 [CAPH2] in condensin), which helps to close the ring. In the case of cohesin in yeast, the Smc3–Rad21 interface has been proposed to form an exit gate for DNA from the ring [4]. The proposed entry and exit of DNA from the cohesin ring is likely a highly regulated process [4]. It is not known whether other SMC complexes such as condensin are similarly gated for DNA entry and exit. ATP hydrolysis is crucial for functional association of SMC complexes with chromosomes [5]. Additional subunits are also associated with the ring, depending on the complex (stromal antigen [SA] for cohesin and non-SMC condensin-associated protein D [NCAPD] and non-SMC condensin-associated protein G [NCAPG] subunits for condensin).

SMC complexes associate with DNA but do not appear to contain specificity for particular DNA sequences. Their association with specific sites on chromosomes may depend on their interaction with additional proteins that can bind to specific DNA sequences or histone modifications. We refer to these as adaptor proteins below.

## SMC complexes and genome organization

The cohesin complex is perhaps the best studied of the SMC complexes. Cohesin is thought to bring two double-stranded DNAs into close physical proximity by topological entrapment, creating cohesion between sister chromatids [7]. Complete loss of a cohesin subunit is lethal, causing dissolution of the cohesion between sister chromatids and precocious separation in metaphase [8], ending in an anaphase with inaccurate chromosome segregation and aneuploidy. However, partial loss of cohesion activity is tolerated but results in a variety of effects on gene expression in interphase. These effects are thought to stem from failed looping events or DNA–DNA interaction events between nonadjacent sequences that may depend on the cohesin complex [9]. For example, if cohesin normally facilitates interaction between a promoter and an enhancer in a particular cell type, loss may result in reduced gene expression if the loop fails to form. However, if cohesin normally facilitates interaction between a promoter and a repressive element, loss may result in increased expression if the loop fails to form. Therefore, the context dependence of the interaction is important to understand the functional outcome.

The cohesin complex is important for interactions between distant sequences at different scales. Promoter–enhancer loops are usually measured in kb. However, at a larger sub-megabase scale, genomes are organized into topological associated domains (TADs) [10]. These domains are defined by Hi-C data collected by genome-wide chromosome conformation capture methods, which reveal regions in which interactions within a domain are substantially higher than interaction with neighboring domains [10]. Both the DNA loops within the TADs as well as the domains themselves are dependent to varying extents on cohesin [11–17]. The deletion of a boundary region between two domains can affect the formation of surrounding topological domains and gene expression, with pathological consequences for development [18]. For example, an enhancer in one domain may normally be restricted to acting on a promoter within the domain but can act on a promoter in a neighboring domain if the boundary between the domains is lost, driving inappropriate gene expression.

The positions of the boundaries between TADs are evolutionarily conserved between the mouse and human genomes [19] and tend to contain many genes (gene dense), and the genes themselves are highly expressed [10]. Many proteins are found at boundaries. In the *Drosophila melanogaster* genome, boundaries contain many instances of binding of several architectural proteins, including the condensin subunits [20]. Consistent with the idea that SMC complexes are often present at transcriptionally active regions, cohesin and condensin complexes are both present at transcriptional regulatory elements in mouse embryonic stem cells [21]. Without cohesin, topological domains largely dissolve [11–16], suggesting that the cohesin complex may be one of the main protein complexes responsible for maintenance of this level of chromosome organization.

Another level of chromosome organization has been termed compartments. Compartments reflect the organization of the nucleus into zones of active/euchromatin and inactive/heterochromatin chromatin. While loops and TADs depend on cohesin [11–17], the proteins responsible for maintaining transcriptionally active and inactive compartments are unclear. The boundary–boundary interactions that form compartments may be mediated by the architectural proteins found at these regions. For example, these regions in mammalian cells contain high numbers of enhancers (superenhancers), and interactions between these domains could potentially form transcription hubs [17]. Heterchromatic compartments may also form based on their protein content; heterochromatin protein 1α (HP1α) may play a role in the phase separation of inactive compartments [22].

**Fig 2:**
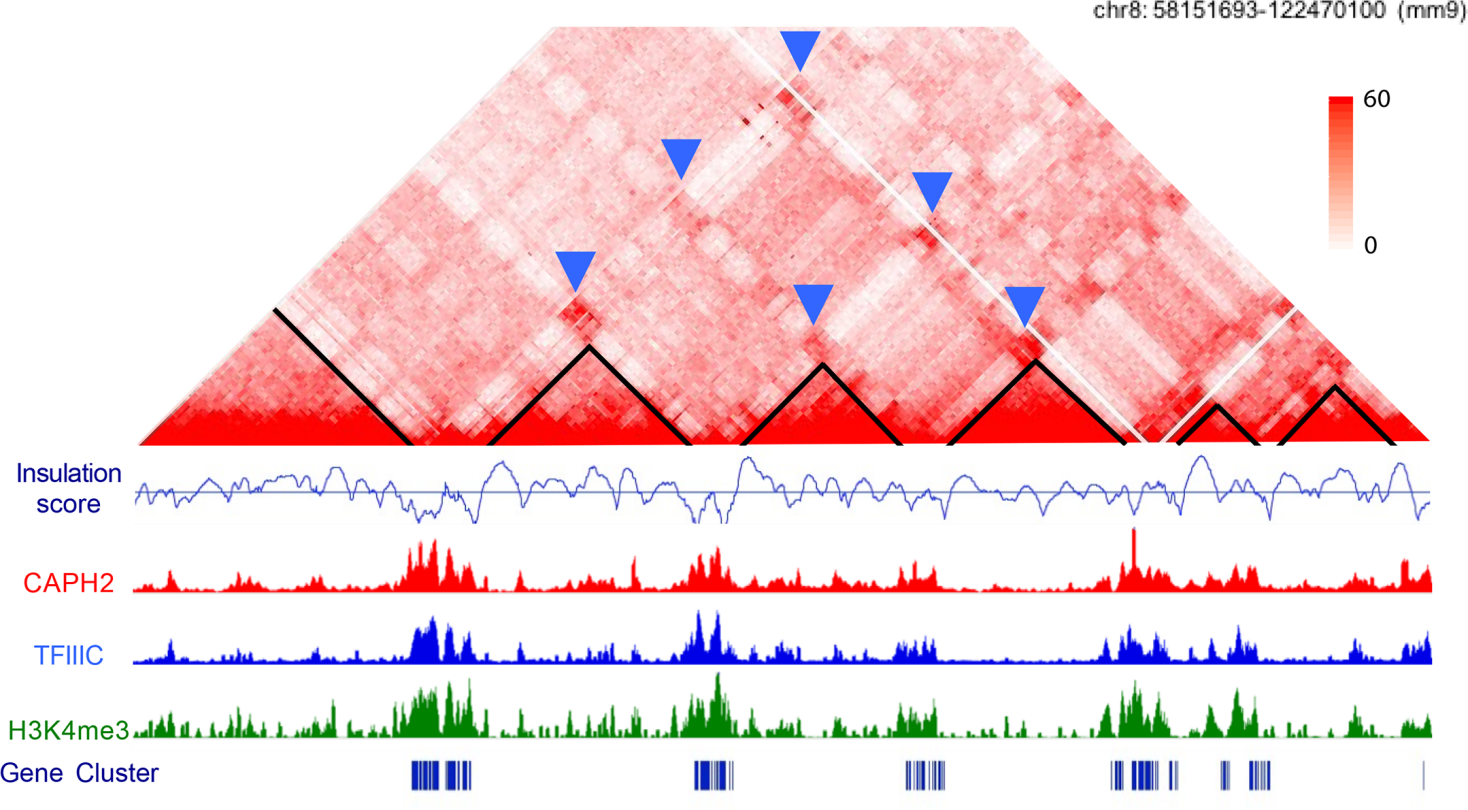
Condensin II and TFIIIC binding co-occur at active gene clusters at TAD boundaries that interact to form compartments. CAPH2, condensin-associated protein H2; TAD, topological associated domain; TFIIIC, Transcription Factor IIIC. An amalgam of data is shown for a region on mouse chromosome 8 (coordinates 58151693–122470100). In the Hi-C contact map [10], TADs are outlined in black. The insulation score, calculated in-house based on the method described in [24], helps to define TADs and boundary regions. Chromatin immunoprecipitation followed by deep sequencing (ChIP seq) data are shown for NCAPH2 [21], a subunit of condensin II, TFCIII90 subunit of TFIIIC [25], H3K4me3 [26], a marker of active promoters, and gene clusters. The blue arrows in the contact map indicate interactions between boundary domains, which are thought to form compartments.

Interestingly, condensin and Transcription Factor IIIC (TFIIIC) complexes colocalize at sites that have boundary–boundary interactions in Hi-C data, suggesting the possibility that condensin II may, in some instances, promote compartment interactions (Fig 2). Like superenhancers, multiple binding sites for TFIIIC and condensin II complexes can be present in a single boundary region. The insulation score method to calculate boundary strength divides the genome into bins, followed by calculating the average of the interaction frequencies across each of the neighboring bins. The insulation scores for strong TAD boundaries will be lower than those for weak boundaries because the average number of interactions across the bins is lower. The insulation scores are higher with fewer condensin II–TFIIIC sites, and lower with more sites, suggesting that densely clustered sites occur at the strongest boundaries [23]. Most of the genes that show down-regulation with condensin subunit knockdown are highly expressed genes located at boundary regions, including the histone genes [23]. Furthermore, the interaction of the histone gene clusters, which occur at boundaries between domains, depends on condensin II, supporting the idea that condensin may help support the interactions between some highly transcribed regions. The experimental data obtained to date are most consistent with cohesin maintaining loops and TADs, with the possibility of some combination of condensin II and superenhancers facilitating the formation of active compartments and of HP1α facilitating the interactions between inactive compartments. There may be multiple protein factors playing overlapping and complementary functions in the formation of compartments.

The definition of TADs and compartments is based largely on chromosome conformation capture data [27] such as Hi-C, which relies on the technique of detecting formaldehyde crosslinking of interacting regions of DNA. However, features of chromosome morphology and organization were first described nearly 150 years ago. For example, banding patterns of chromosomes were first observed in the early 1880s by Balbiani and Flemming. The new concepts of TADs and boundaries based on Hi-C data turn out to correspond with the long-ago observed bands and interbands of polytene chromosomes of *D*. *melanogaster* as determined by careful comparison between microscopy and Hi-C methods [28]. Another observation made independently by Rabl and Boveri around the turn of the century is that chromosomes occupy particular regions of the nucleus in interphase, termed chromosome territories by Boveri. Condensin II promotes the formation of chromosome territories in *D*. *melanogaster* [29, 30]. An important goal for the future will be to reinterpret classic observations regarding chromosome morphology and organization in light of new chromosome conformation capture and imaging technologies to understand how chromosomes are organized in the nuclear space at different levels. This will allow an enhanced appreciation for the role of SMC complexes in chromosome organization and morphology.

## Adaptor proteins for SMC complexes

Although SMC complexes associate with particular regions of DNA reproducibly in chromatin immunoprecipitation experiments, they do not appear to possess specificity for any particular sequence. Sequence-specific binding is conferred in some instances by associated proteins or protein complexes and histone modifications (Table 1). The association of cohesin with chromatin depends on the sequence-specific CCCTC-binding factor (CTCF) at many locations that contain the CTCF consensus binding sequence [31–33]. However, in yeast, it is not clear if there is any sequence specificity to cohesin binding because yeast lack CTCF, and the Scc2-Scc4 loading complex does not appear to have sequence specificity. In addition, nematodes have lost CTCF [34], and cohesin and CTCF do not strongly colocalize in the *D*. *melanogaster* genome, making use of CTCF as an adaptor restricted to fish and mammals. However, transcriptional activity is a unifying theme in the localization of the cohesin complex in eukaryotes [9].

**Table 1:** SMC complexes, adaptor proteins, and associated histone modifications.

Complex	Organism	DNA site	Sequence recognizer	Epigenetic marks
SMC	*Bacillus subtilis*	*parS*	ParB (31,32)	NA
Condensin	Yeast/mouse/human	B box	TFIIIC (22,37)	H3K4me3 (22)
				H4K20me1 (44)
	Flies		Mrg15 (52,53)	acetylated lysines on histone tails
	Mammals/flies		pRB (54)	
	Mammals		Brd4 (51)	methylated lysines on histone tails
Cohesin	Mouse/human	CCCTC	CTCF (27,28)	unknown
Dosage compensation	*C*. *elegans*	rex	unknown	H4K20me1 (45,46)

Abbreviations: NA, not applicable; SMC, structural maintenance of chromosome.

The sequence-specific adaptor proteins for the DCC and Smc5/6 are much less well understood, but the DCC in *C*. *elegans* associates with specific sequences known as recruitment elements on X (rex) sites [35]. The bacterial *B*. *subtilis* SMC complex is loaded at a specific sequence, *parS*, by the *parS* binding protein ParB [36, 37]. In some ways, ParB may be more analogous to Nipbl-Mau2 (Scc2-Scc4 in yeast), cohesin complex loading proteins, but the ParB/*parS* system also demonstrates a sequence-specific adaptor protein for an SMC complex in prokaryotes. Several adaptors have been identified for the eukaryotic condensin complex, as discussed below.

There are two types of condensin complexes in the mammalian genome [38], distinguished by their non-SMC subunits. While condensin I is present on chromosomes following nuclear envelope breakdown [39], condensin II is present during interphase [39], making it a likely candidate for an interphase organizer. Newly published work shows that mammalian condensin II, like yeast condensin [40], physically interacts with the TFIIIC complex and furthermore, the two significantly colocalize in several genomes [20, 23, 41]. TFIIIC, like CTCF, binds to a unique sequence (B box) that appears to help anchor condensin II to specific locations in the mammalian genome. In budding yeast, the B box is essential for association of TFIIIC and condensin with a tRNA gene [41]. Furthermore, the colocalization of tRNA genes in three dimensions (3D) is dependent on condensin and TFIIIC in budding and fission yeast [40, 42, 43], strongly suggesting a role in the organization of chromosomes in the nuclear space.

TFIIIC is a multi-subunit transcription factor protein complex for RNA polymerase III, but there are about 10 times more locations in the mammalian genome at which TFIIIC binds than RNA pol III [44–46]. A significant fraction of these extra sites may participate in organization of the mammalian genome. Knockdown of a subunit of TFIIIC resulted in a lower ChIP signal for condensin II, but the reverse was not true, consistent with the idea that TFIIIC binds to the genome based on its binding site, and the condensin complex binds at these sites based on its association with the TFIIIC complex. These data are the first to suggest that the TFIIIC complex may be involved in anchoring the condensin II complex to specific locations in the mammalian genome. TFIIIC may be an evolutionarily conserved adaptor protein for condensin from yeast to human.

In conjunction with sequence-specific binding proteins, some histone marks also appear to participate in determining SMC binding regions, although condensins also show enrichment at nucleosome-depleted regions [47]. For example, condensin II subunits are capable of recognizing H4K20me1 and significantly overlap with sites with this histone mark in HeLa cells [48]. This same mark is associated with the DCC in *C*. *elegans* [49, 50]. The NCAPD3 subunit of condensin II binds to a peptide of H3 with lysine 4 trimethylation, and furthermore, the condensin II complex, the TFIIIC complex, and H3K4me3 significantly colocalize in the mouse and human genomes [23]. Some of these locations are boundary regions between TADs that (1) contain multiple instances of binding sites, similar to the high-density architectural protein binding sites defined in the *Drosophila* genome [20], and (2) show compartment interactions.

Interestingly, the loading of the centromeric histone variant centromere protein A (CENP-A) depends on an interaction between the condensin complex and Holliday junction recognition protein (HJURP), the centromeric histone chaperone [51]. At centromere regions, the condensin complex may help to establish centromeric chromatin, and its loss may compromise centromere function [52–54]. Similarly, loss of cohesin activity impacts chromosomal arm regions and centromeric regions differently, with loss at arm regions generally impacting chromosomal organization and gene expression and loss at centromere regions negatively impacting chromosome segregation. This suggests that different adaptor proteins may interact with SMC complexes at different locations in the genome, guiding association and function. Additional examples of adaptor proteins for the condensin complex include the bromodomain protein Brd4 [55], which can modulate the signaling response to DNA damage by recruiting condensin, chromodomain protein MORF-related gene (Mrg15), which promotes maintenance of interphase chromosome compaction and homolog pairing [56, 57], and retinoblastoma protein (pRB), which promotes condensation [58]. These many adaptors for the condensin complex begin to suggest that loss of condensin activity, like loss of cohesin activity, may have cell and chromosome context-specific effects. As epigenetic marks and sequence-specific guiding proteins may influence SMC complex association with the genome, these factors then have the potential to influence chromosome organization. It remains to be determined what additional proteins or epigenetic marks help guide SMC complexes to specific locations in the genome.

## SMC complexes and loop extrusion

**Fig 3:**
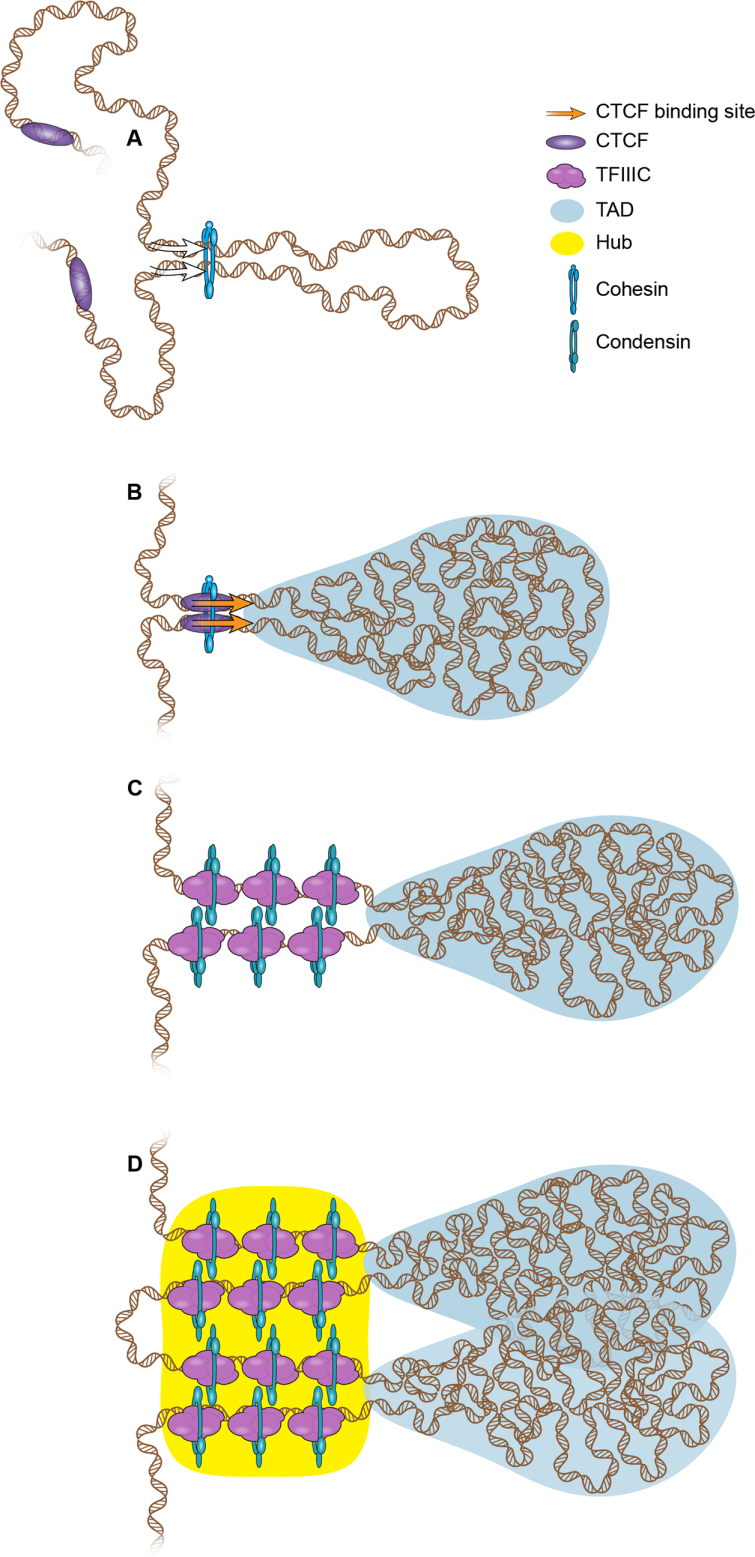
Working models for how adaptor proteins and SMC complexes facilitate the formation of chromosomal domains by loop extrusion. (A) A loop begins to form as DNA is extruded through the center of an SMC complex ring (blue). Protein rings may move along DNA until an adaptor protein (purple) is encountered that captures the SMC complex ring and halts the extrusion. (B) A region is depicted with cohesin and CTCF facilitating the formation of a topological domain by constraining the sequences at the base. CTCF sites tend to be arranged head-to-head [62]. A single cohesin ring may interact with CTCF and encircle two DNAs to form the base of the loop, which is the boundary between neighboring TADs. Alternatively, two cohesin rings may interact to facilitate the DNA–DNA interaction. (C) Multiple binding sites for condensin II and TFIIIC are found at TAD boundaries. It is not known whether a single condensin ring can encircle two DNAs; we have depicted one ring per DNA, similar to the handcuff model proposed for the bacterial SMC complex in loop extrusion. (D) Interactions between active genes at the base of multiple domains may facilitate the high levels of expression of housekeeping genes, perhaps via a local transcriptional hub (yellow). CTCF, CCCTC-binding factor; SMC, structural maintenance of chromosome; TAD, topological associated domain; TFIIIC, Transcription Factor IIIC.

Because SMC complexes have evolutionarily conserved structural similarities, it seems likely that they will also organize DNA using similar mechanisms. One currently popular model proposed to explain how SMC complexes organize chromosomes is the loop extrusion model (Fig 3) [59]. In this model, DNA moves through the lumen of the SMC protein complex, processively extruding a loop [60]. The condensin complex is a mechanochemical motor that translocates along DNA, consistent with the ability to extrude loops [61]. In the case of the cohesin–CTCF pair, CTCF sites oriented toward each other may halt cohesin and specify the base of the loop [19, 62, 63]. Loop extrusion has been proposed to underlie TAD formation [64], and recent experiments show that loops are lost when cohesin is degraded but reform quickly once it is restored, suggesting that cohesin may be responsible for extruding loops that constitute TADs. Interestingly, compartments are not lost upon cohesin loss, suggesting that additional proteins could regulate this level of chromosome organization. How the activities of cohesin and condensin are similar and distinct in terms of loop extrusion is an open question.

One prediction of the loop extrusion theory is that multiple complexes may become trapped at the base of the loop [60]. Multiple instances of particular proteins, such as those present at superenhancers at the boundaries of TADs, may facilitate interactions between boundary domains to form active compartments. It is important to recognize that TADs are based on population averages, and any single cell might not have the “average” TAD structure [11]. Because multiple TFIIIC binding sites correlate with stronger insulation and boundaries between domains, multiple TFIIIC sites could increase capture frequency for the condensin complex, creating a stronger boundary. Continuing efforts of mathematical modeling, single-cell analysis, imaging, and genome-wide analysis will help to resolve the organizational roles of different SMC complexes and additional proteins.

**Fig 4:**
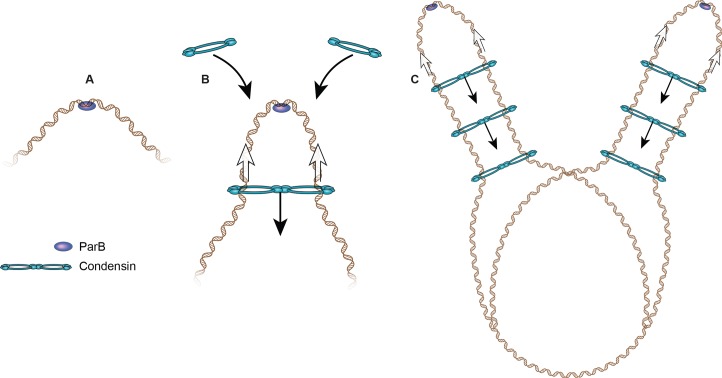
Proposed loop extrusion model for how the SMC complex in *B*. *subtilis* mediates the interaction between chromosome arms. (A) Prior to condensin loading, ParB (purple) binds to a *parS* site. At this point, there is no interaction between chromosome arms. (B) The condensin complex (blue) loads at *parS* sites dependent on the ParB protein, and then “handcuffs” can move along DNA (black arrows), juxtaposing the two sides of the chromosome. (C) As more condensin loads at the *parS* site and spreads away from the site, and DNA replicates, the arms of the chromosomes are zipped up. Given the structural similarities between SMC complexes from bacteria and eukaryotes, it seems likely there will be common themes by which SMC complexes organize DNA. Cartoon adapted from [65]. ParB, Partition B; SMC, structural maintenance of chromosome.

Recent evidence that an SMC complex may organize DNA via loop extrusion comes from *B*. *subtilis* [65, 66]. The Rudner lab used an inducible system to show that the bacterial condensin complex, loaded at a *parS* site adjacent to the replication origin, travels down the left and right chromosome arms while tethering them together, in essence extruding a single large (4 Mb) DNA loop centered on the origin. The rate of travel is greater than 50 kb/min, and the movement of condensin scales linearly with time, suggesting an active transport model. The condensin rings may travel as a joined pair, sometimes referred to as a hand-cuff model (Fig 4) [67]. Interestingly, highly transcribed genes and other obstacles appear to reduce the rate of movement through a region, but the condensin complex ultimately traverses these obstacles.

The reintroduction of cohesin in mammalian cells reveals a rate of loop formation on par with *B*. *subtilis* [17, 65]. These results suggest that if loop extrusion is a common mechanism by which SMC complexes organize chromosomes, the SMC complexes in prokaryotes and eukaryotes may operate with similar rates. In mammalian cells, particular adaptors proteins such as CTCF or the TFIIIC complex may “capture” SMC complexes, forming the base of a loop or domain instead of simply slowing the complex. SMC complexes may be found at higher frequency at transcriptionally active regions due to the presence of “capture” proteins but also based on the intrinsic ability of these regions to act as impediments to movement as demonstrated in prokaryotes. Loop extrusion is an attractive model because it suggests how SMC complexes could arrange large domains of chromatin into loops that are topologically distinct from each other.

## SMC complexes in human health and disease

In addition to locus-specific effects, the condensin complex may have cell type–specific effects. Mutations in genes encoding condensin subunits cause microcephaly [68] and cancer [69, 70]. In these instances, loss of condensin activity leads to defects in chromosome segregation, which include anaphase bridges, micronuclei, and aneuploidy. In the case of microcephaly, the proposed model is that loss of condensin activity in neural stem cells leads to chromosome instability and compromises cell proliferation and survival, leading to a smaller brain. Mutations in microcephalin 1 (*MCPH1*), a gene encoding a negative regulator of condensin II, also cause microcephaly, suggesting that both loss of condensin function and overactive condensation can both negatively impact brain development and cause microcephaly [71]. In a mouse model for T-cell lymphoma, a mutation in a gene encoding a subunit of the condensin complex confers differential ploidy maintenance depending on the hematopoietic cell type, with the most compromised CD4^+^/CD8^+^ T cells being those from which the cancer initiates [70]. One challenge is to reconcile the role of the condensin complex in the structural maintenance of chromosomes with its causal role in these diseases. The molecular explanation will likely require that we consider both chromosomal context and cell type.

Phenotypes occurring due to loss of condensin function may be an amalgam of affected chromosomal processes. For example, highly expressed regions may lose expression if condensin normally promotes the coalescence of genes into compartments or hubs with high transcriptional activity. This could affect the ability to maintain a gene expression program—thereby compromising the creation or maintenance of specific cell states and tissues—or create enhanced search space for new gene expression programs [72]. Chromosomal locations at which condensin helps to maintain a specialized nucleosome, such as centromeres, may have compromised chromatin, affecting chromosome segregation. Therefore, condensin could promote the generation and maintenance of specific cell types and tissues via multiple mechanisms. Future efforts aimed at comprehensive analysis of the basic molecular functions of SMC complexes in different cell types will improve our understanding of the molecular etiology of diseases caused by mutations in genes encoding the subunits of condensin.

## Conclusions

SMC complexes are ancient protein complexes with conserved functions in organizing and maintaining genomes. SMC complexes have important roles in human development and cancer. Recent studies have uncovered that their localization is often regulated by adaptor proteins that can recognize specific DNA sequences or modified histones. Current models postulate that SMC complexes organize the genome into loops or domains via extrusion. Extrusion may halt at adaptor proteins such as CTCF for cohesin. The molecular basis of compartment formation is not currently clear but could be protein based. We must continue to study how SMC complexes contribute to chromosome organization, gene expression, and chromosome transmission in different contexts to understand how they shape and maintain genomes.
